# A_2A_ adenosine receptors are involved in the reparative response of tendon cells to pulsed electromagnetic fields

**DOI:** 10.1371/journal.pone.0239807

**Published:** 2020-09-30

**Authors:** Alessandra Colombini, Carlotta Perucca Orfei, Fabrizio Vincenzi, Paola De Luca, Enrico Ragni, Marco Viganò, Stefania Setti, Katia Varani, Laura de Girolamo

**Affiliations:** 1 Orthopaedic Biotechnology Lab, IRCCS Istituto Ortopedico Galeazzi, Milan, Italy; 2 Department of Morphology, Surgery and Experimental Medicine, University of Ferrara, Ferrara, Italy; 3 IGEA SpA, Carpi, Modena, Italy; Universite Paris-Sud, FRANCE

## Abstract

Tendinopathy is a degenerative disease in which inflammatory mediators have been found to be sometimes present. The interaction between inflammation and matrix remodeling in human tendon cells (TCs) is supported by the secretion of cytokines such as IL-1β, IL-6 and IL-33. In this context, it has been demonstrated that pulsed electromagnetic fields (PEMFs) were able to reduce inflammation and promote tendon marker synthesis. The aim of this study was to evaluate the anabolic and anti-inflammatory PEMF-mediated response on TCs in an *in vitro* model of inflammation. Moreover, since PEMFs enhance the anti-inflammatory efficacy of adenosine through the adenosine receptors (ARs), the study also focused on the role of A_2A_ARs. Human TCs were exposed to PEMFs for 48 hours. After stimulation, A_2A_AR saturation binding experiments were performed. Along with 48 hours PEMF stimulation, TCs were treated with IL-1β and A_2A_AR agonist CGS-21680. IL-1Ra, IL-6, IL-8, IL-10, IL-33, VEGF, TGF-β1, PGE_2_ release and *SCX*, *COL1A1*, *COL3A1*, *ADORA2A* expression were quantified. PEMFs exerted A_2A_AR modulation on TCs and promoted *COL3A1* upregulation and IL-33 secretion. In presence of IL-1β, TCs showed an upregulation of *ADORA2A*, *SCX* and *COL3A1* expression and an increase of IL-6, IL-8, PGE_2_ and VEGF secretion. After PEMF and IL-1β exposure, IL-33 was upregulated, whereas IL-6, PGE_2_ and *ADORA2A* were downregulated. These findings demonstrated that A_2A_ARs have a role in the promotion of the TC anabolic/reparative response to PEMFs and to IL-1β.

## Introduction

Tendinopathy has been considered for many years as a purely degenerative disease devoid of inflammation. Nevertheless, convincing evidences suggested that the inflammatory reaction is a crucial step of the onset and duration of this condition [1, 2], but it also has a role in tendon healing. Soluble inflammatory mediators, such as cytokines and complements, are involved in inflammatory responses in tendinopathy [3]. In particular, the healing of tendon degenerative changes is a complex process that is firstly supported by the secretion of inflammatory cytokines such as IL-6 and IL-1β [4]. These mediators originate both from the inflammatory cells that infiltrate the damaged tissue and from resident tendon cells (TCs), for example in response to mechanical stretching [5, 6]. IL-1β entails the production of inflammatory COX‑2, PGE_2_ and catabolic MMP-1, MMP-3 [7, 8] and increases the ratio of collagen type III/I [9] in human TCs, suggesting an interaction between inflammation and matrix degradation/alteration in tendinopathy. Moreover, IL-1β acts on the phenotype of tendon progenitor cell by strongly decreasing the expression of tenogenic markers scleraxis and tenomodulin [10].

IL-6 expression is increased in pathological tendon [11, 12] and is involved in type I collagen synthesis [13]. Finally, among the most involved secretory cytokines, IL-33 represents an early tissue mediator that is involved in the type III collagen synthesis, in the tissue remodeling and in the maintenance of the balance between reparative and degenerative processes [14].

*In vivo* it was observed that Pulsed ElectroMagnetic Fields (PEMFs) promoted tendon healing through a reduction of inflammation, improvement of mechanical properties and a promotion of better collagen alignment, thus suggesting a reparative role of these stimuli on the degenerative and inflammatory processes involved in tendinopathy [15, 16].

The healing potential of PEMFs has been observed also in an *in vitro* model of tendon lesion, where the exposure to biophysical stimulation significantly accelerated the cut closure time after injury [17, 18].

A dose-dependent response of human TCs to PEMFs (1.5 mT, 75Hz) was observed in previous studies [19, 20], with increase of proliferation, up-regulation of the tissue-specific markers *SCX* and *COL1A1* and angiogenic factor *VEGFA* expression, as well as release of IL-1β, IL-6, IL-10 and TGFβ along with reduced *COL3A1* expression [20].

Under inflammatory conditions (10 ng/ml of IL-1α), 3 hours/day of PEMF treatment promoted upregulation of *COL1A1* at 1 week and of *TGFB1*, PDGFB, *BMP12* and *TIMP4* at 2 weeks in human TCs [21]. Moreover, in rat TC-derived 3D constructs the exposure to high-energy PEMF treatment in inflamed condition (10 ng/mL of IL-1β) affected several biological processes such as extracellular matrix remodelling, inflammation and negative regulation of apoptosis [22]. Adenosine, interacting with A_1_, A_2A_, A_2B_ and A_3_ adenosine receptors (ARs) plays an important role in different pathologies based on inflammation status exerting selective potential protective effects [23, 24]. Moreover, it has been also reported that PEMFs enhance the efficacy of endogenous adenosine as an anti-inflammatory agent through the AR upregulation [25, 26]. PEMFs were also able to modulate cartilage and bone metabolism in human synoviocytes, chondrocytes, and osteoblasts showing various positive effects at articular level [27]. In particular, PEMFs treatment determined a significant upregulation of A_2A_ and A_3_ ARs with reduction of the release of some pro-inflammatory cytokines such as PGE_2_, IL-6 and IL-8 [25, 26, 28].

Given these premises, in this study the possible anabolic and/or an anti-inflammatory PEMFs-mediated response on TCs has been evaluated in an *in vitro* model of early tendinopathy (inflammatory phase). The novelty of the present investigation is the attempt to clarify the role of A_2A_ARs in the response of TCs to PEMF stimulation in this experimental setting. The findings of this study will be helpful in the understanding of the links between the inflammatory mechanisms involved in tendon healing and the tissue damage, and in the selection of possible pharmacological targets for the development of novel specific therapies for tendinopathies.

## Materials and methods

### TCs isolation and culture

The study was reviewed and approved by IRCCS Istituto Ortopedico Galeazzi IRB. Waste surgical samples of semitendinosus and gracilis tendons were collected with the written informed consent of patients (M-SPER-015- Ver. 2–04.11.2016 for the use of surgical waste material) from five donors (males, age range 14–44 years) who underwent anterior cruciate ligament reconstruction.

After 16 hours of enzymatic digestion with 0.3% w/v type I collagenase (Worthington Biochemical Corporation, Lakewood, NJ, USA) [19], the tendon samples were filtered through a 100 μm cell strainer (Becton, Dickinson and Co., NJ, USA) and centrifuged (300 g, 5 minutes). The resulting TCs were plated at a density of 5x10^3^ cells/cm^2^ in control medium composed of Dulbecco's Modified Eagle Medium High Glucose (SIGMA Aldrich), 10% fetal bovine serum (FBS, Sigma-Aldrich, St. Louis, MO, USA), 50 U/mL penicillin, 50 mg/mL streptomycin, 2 mM L-glutamine (Life Technologies) and maintained in incubator at 37°C in humidified atmosphere with 5% CO_2_. TCs were cultured until passage 3, when they were detached and seeded for the following experiments.

### PEMF stimulation

After 24 hours from the seeding, TCs were exposed to PEMF stimulation. The electromagnetic field was generated by a pair of rectangular horizontal coils (18 x 13 cm), each made of 1,000 turns of copper wire, placed opposite each other, directly inside the incubator, for a duration of 48 hours. The coils were powered by a PEMF generator system (IGEA, Carpi, Italy), with a magnetic field intensity of 1.5 mTesla (1.5mT) and a frequency of 75 Hz, yielding a 10% duty cycle, as already used in previous studies [19, 20]. The physical parameters of PEMFs were kept constant through the exposure time and during the experiments. Unstimulated cells were used as control.

### A_2A_AR saturation binding experiments

Cell fractions obtained from 2x10^6^ cells stimulated or not with PEMF for 48 hours were centrifuged in hypotonic buffer at 20,000 x g for 10 min to obtain a membrane suspension for A_2A_AR saturation binding experiments. The resulting pellet was suspended in Tris HCl 50 mM buffer pH 7.4 with 2 IU/mL adenosine deaminase (Sigma-Aldrich) and incubated for 30 min at 37°C. After a centrifugation at 40,000 x g for 10 min, the final pellet was used for radioligand binding assays. The protein concentration was determined by a Bio-Rad method with bovine albumin as reference standard. Saturation binding experiments to A_2A_ARs was carried out by using different concentrations (0.01–30 nM) of ^3^H-ZM 241385 as radioligand and cell membranes (60 μg) that were incubated for 60 min at 4°C [28]. The radioligand ^3^H-4-(2-(7-amino-2-(2-furyl)(1,2,4)-triazolo (2,3-a)(1,3,5) triazin-5-ylamino) ethyl) phenol (^3^H-ZM 241385; specific activity, 27 Ci/mmol) was purchased from Biotrend, Cologne, Germany. Non-specific binding was determined in the presence of ZM 241385 1 μM. Bound and free radioactivity were separated by filtering the assay mixture through Whatman GF/B glass fiber filters by using a Brandel cell harvester [28]. The filter bound radioactivity was counted in a 2810 TR liquid scintillation counter Packard (Perkin Elmer Life and Analytical Sciences, USA).

Three technical replicates were analyzed for each experimental group.

### Treatment with pro-inflammatory stimulus and A_2A_AR agonist

Along with 48 hours PEMF stimulation, 2x10^5^ TCs were treated by 1 ng/ml of IL-1β or 1 μM of A_2A_AR agonist CGS-21680, alone or combined. Untreated cells were used as control. At the end of the 48 hours, the supernatant and the cells were collected and used to evaluate the response to the treatments.

### Gene expression analysis

Total RNA was extracted using PureLink® RNA Mini Kit (Thermo Fisher Scientific, Waltham, MA USA) and quantified spectrophotometrically (NanoDrop, Thermo Fisher Scientific). Purity was estimated as 260/280 nm absorbance ratio and in presence of a ratio of ~2.0 the samples were reverse transcribed to cDNA (5 min at 25°C, 30 min at 42°C and 5 min at 85°C) using a iScript™ cDNA Synthesis Kit (Bio-Rad Laboratories, CA, USA). Real time PCR (StepOne Plus, Thermo Fisher Scientific, Waltham, MA, USA) was performed. Ten ng of cDNA were used as template and incubated with a PCR mix containing TaqMan® Universal PCR Master Mix and Assays-on-Demand Gene expression probes (Life Technologies) for the following genes: *SCX*, Hs03054634_g1, *COL1A1*, Hs01076777_m1, *COL3A1*, Hs00943809_m1 and *ADORA2A*, Hs00169123_m1. Reactions were performed with Applied Biosystems® StepOnePlus™ (Life Technologies; 50°C for 2 min, 95°C for 10 min, 40 cycles at 95°C for 15 s and 60°C for 1 min). The fold change in expression was normalized against the expression of *GAPDH*, Hs99999905_m1, validated as the most stable in this experimental setting. Two technical replicates were analyzed for each experimental group. Data were expressed according to the dCt method.

### Cytokine quantification

IL-10, VEGF and TGF-β1 levels were measured by using the AlphaLISA specific kit (Perkin Elmer Life and Analytical Sciences, USA). Briefly, aliquots of the samples were incubated in presence of specific biotinylated anti-analyte antibody and anti-analyte antibody-conjugated acceptor beads. After incubating for 60 min, streptavidin-coated donor beads were added. Singlet oxygen generated by donor beads excites the acceptor beads, which emit light proportional to the level of interaction. Plates were read with the Perkin Elmer EnSight multimode plate reader (Perkin Elmer Life and Analytical Sciences, USA). The detection range were 4.4–30000 pg/ml for IL-10, 2.2–100000 pg/ml for VEGF and 9–100000 pg/ml for TGF-β1.

PGE_2_ levels were evaluated by a means of a specific ELISA assay following the manufacturer's instructions (R&D Systems, USA). The detection range was 39–2500 pg/ml.

The levels of soluble IL-1Ra, IL-6, IL-8 and IL-33 in cell culture medium were determined by commercially available ELISA assays according to the manufacturers’ instructions (PeproTech, Hamburg, Germany). The detection range were 23–1500 pg/ml for IL-1Ra and IL-6, 16–1000 pg/ml for IL-8 and of 31–4000 pg/ml for IL-33.

Two technical replicates were analyzed for each experimental group.

### Statistical analysis

Dissociation equilibrium constants for saturation binding, affinity or KD values, as well as the maximum densities of specific binding sites, Bmax values were calculated for a system of one or two-binding site populations by non-linear curve fitting using the program Ligand purchased from Kell Biosoft [28]. Kruskal-Wallis test for unpaired data (binding experiments at 48 hours of PEMF treatment) or Friedman test for paired data followed by Dunn’s post test were used to compare the data. p values <0.05 were considered statistically significant. All data are reported as mean ± SEM of independent experiments. All analysis were carried out using R software v3.6.2.

## Results

### PEMFs exerted A_2A_AR modulation on TCs

**Fig 1:**
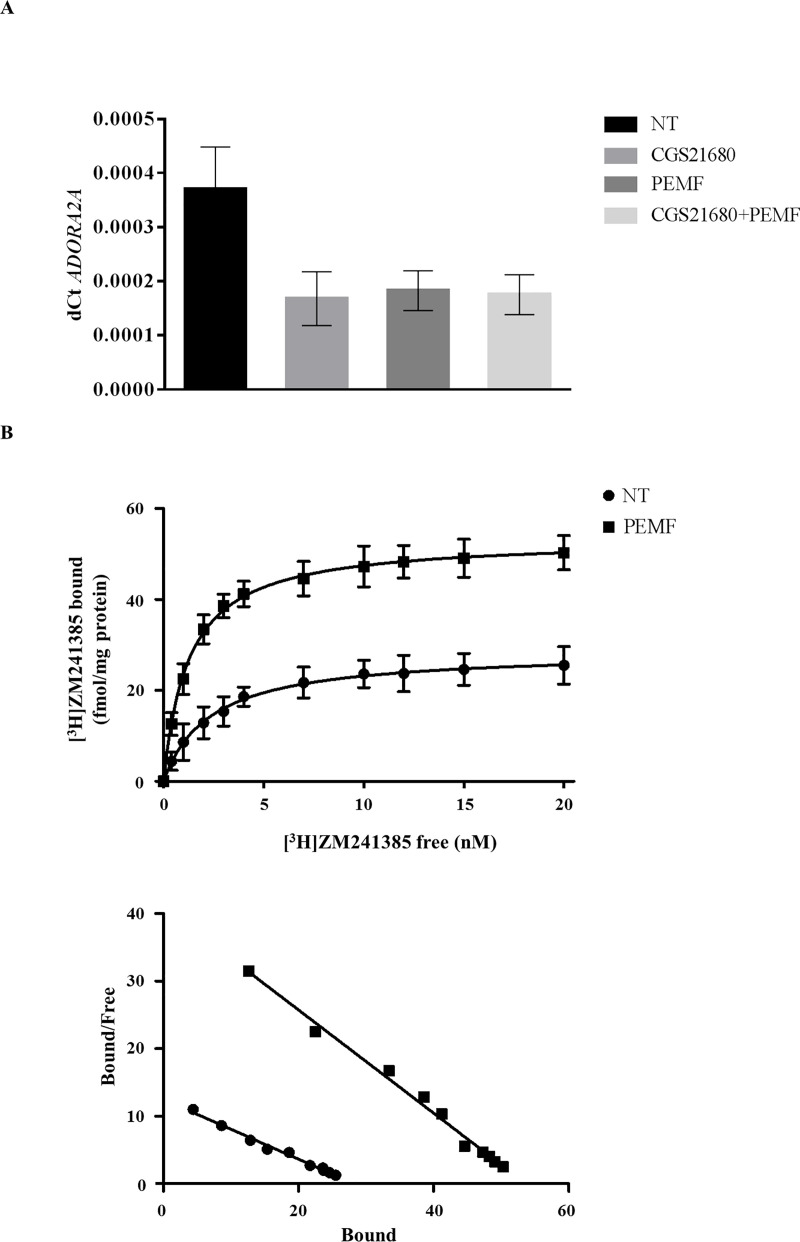
Exposure of TCs to PEMF and CGS-21680 for 48 hours. Expression of *ADORA2A* (A) and A_2A_ adenosine receptor saturation binding experiments (B). n = 5. NT = not treated cells.

The exposure of TCs to PEMFs and CGS-21680 for 48 hours affected both *ADORA2A* expression (Fig 1A) and A_2A_ARs density or ligand affinity (Table 1, Fig 1B), although not significantly.

**Table 1:** Dissociation equilibrium constants for A_2A_AR saturation binding experiments.

	K_D_ (nM)	Bmax (fmol/mg protein)
TCs	2.46±0.09	28.7±1.8
TCs + 48 h PEMFs	1.29±0.10	53.3±3.5
TCs + IL-1β	2.45±0.05	84.4±4.7
TCs + IL-1β + 48 h PEMFs	1.24±0.08	135.8±7.7

K_D_ = affinity values, Bmax = maximum densities of specific binding sites.

### PEMFs represented an anabolic and reparative stimulus on TCs

Forty-eight hours of exposure to PEMFs did not determine cell detachment or different amount of collected RNA after extraction, suggesting no cell death.

**Fig 2:**
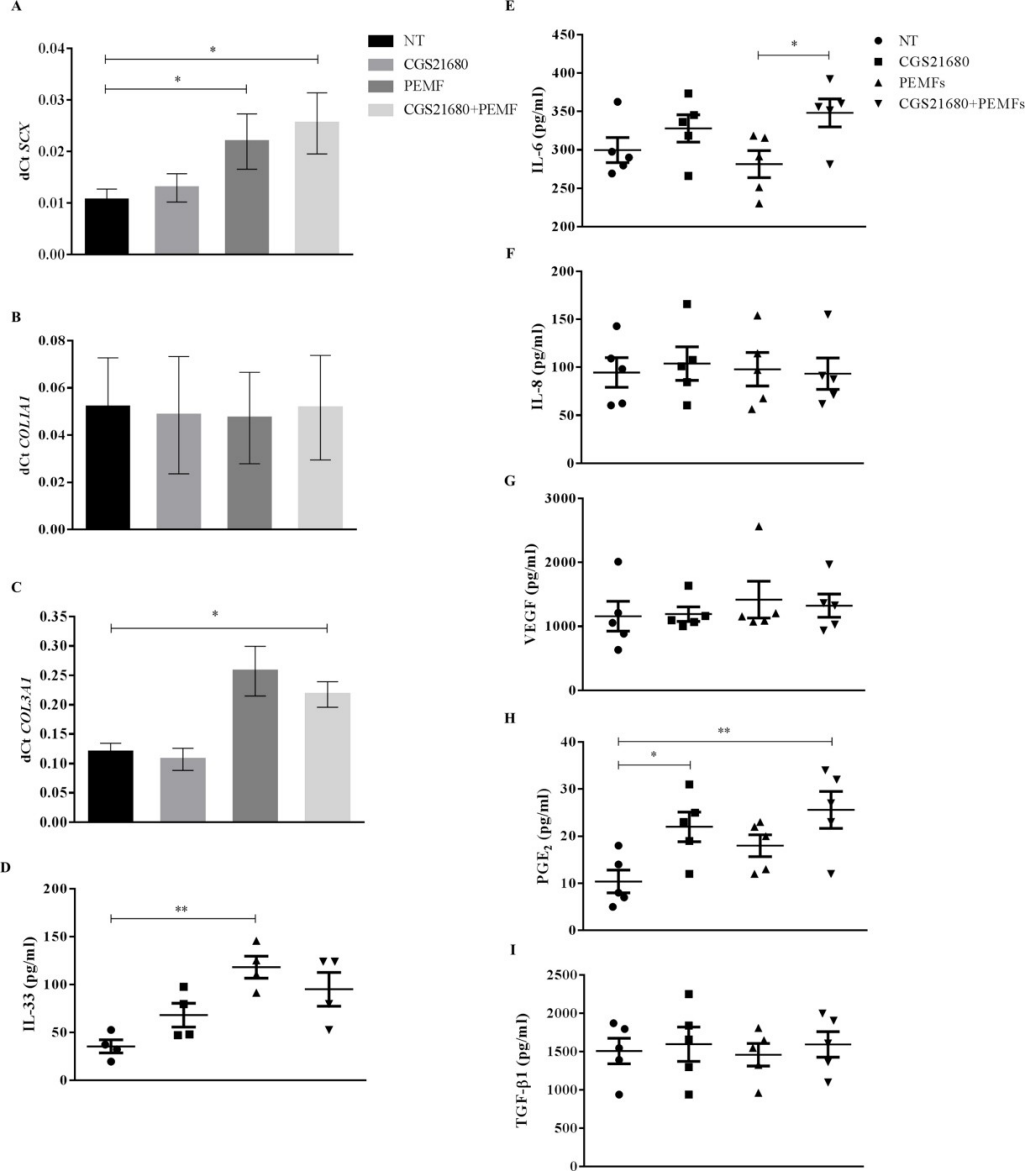
Gene expression and protein release of TCs after 48 hours exposure to PEMF and/or CGS-21680. *SCX* (A), *COL1A1* (B) and *COL3A1* (C) expression, IL-33 (D), IL-6 (E), IL-8 (F), VEGF (G), PGE_2_ (H) and TGFβ (I) secretion. * p<0.05, ** p<0.01. n = 5. NT = not treated cells.

An upregulation of *SCX* in TCs after PEMF treatment, alone or combined with CGS-21680 (p = 0.04 and p = 0.01 *vs* NT, respectively), was observed (Fig 2A). Similarly, an increase of *COL3A1* expression (p = 0.04 for NT *vs* CGS-21680+PEMFs) (Fig 2C), along with an increased IL-33 secretion was also observed (p = 0.004 for NT *vs* PEMFs) (Fig 2D). IL-6 secretion was significantly enhanced by PEMFs but only when combined with CGS-21680 (p = 0.03 *vs* PEMF) (Fig 2E), while PGE_2_ release was promoted by both CGS-21680 alone and combined with PEMFs (p = 0.03 and p = 0.005 *vs* NT, respectively) (Fig 2H). None of the treatments was able to modulate *COL1A1*, IL-8, VEGF and TGFβ (Fig 2B, 2F, 2G and 2I).

### Anabolic and reparative response of TCs to inflammation

**Fig 3:**
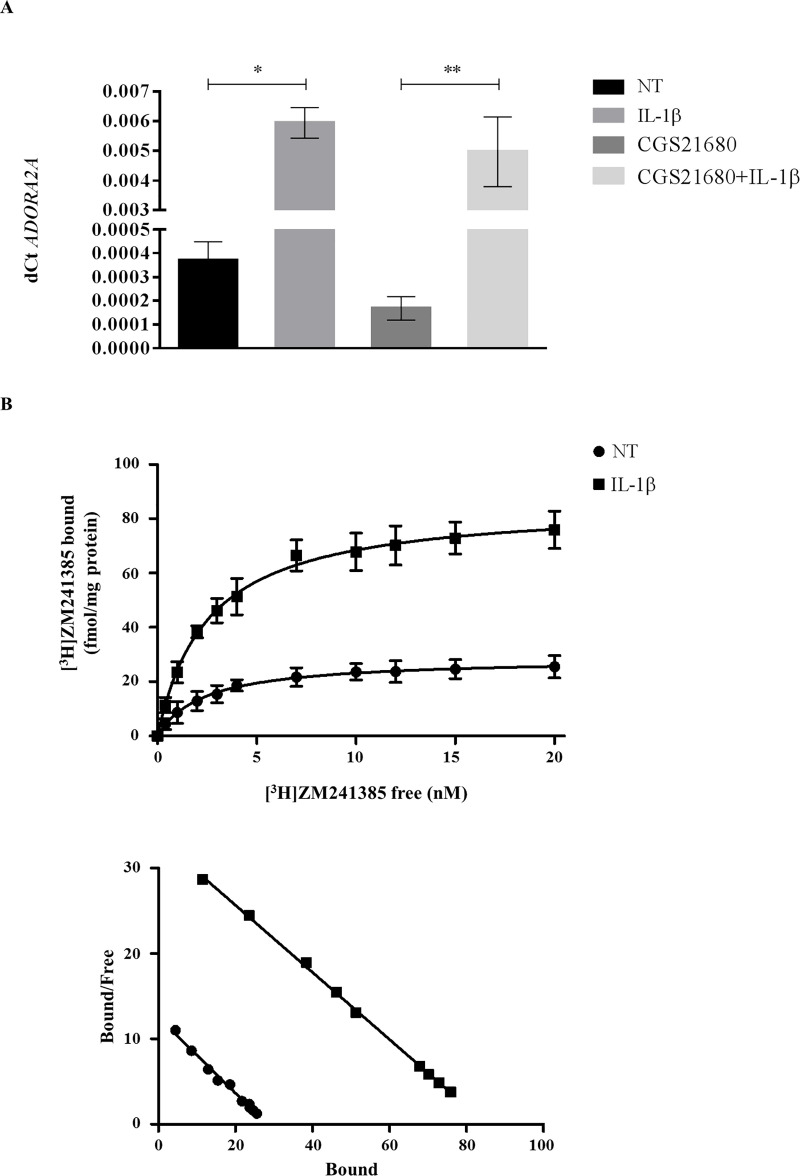
Exposure of TCs to IL-1β and CGS-21680 for 48 hours. Expression of *ADORA2A* (A) and A_2A_ adenosine receptor saturation binding experiments (B). * p<0.05, ** p<0.01. n = 5. NT = not treated cells.

**Fig 4:**
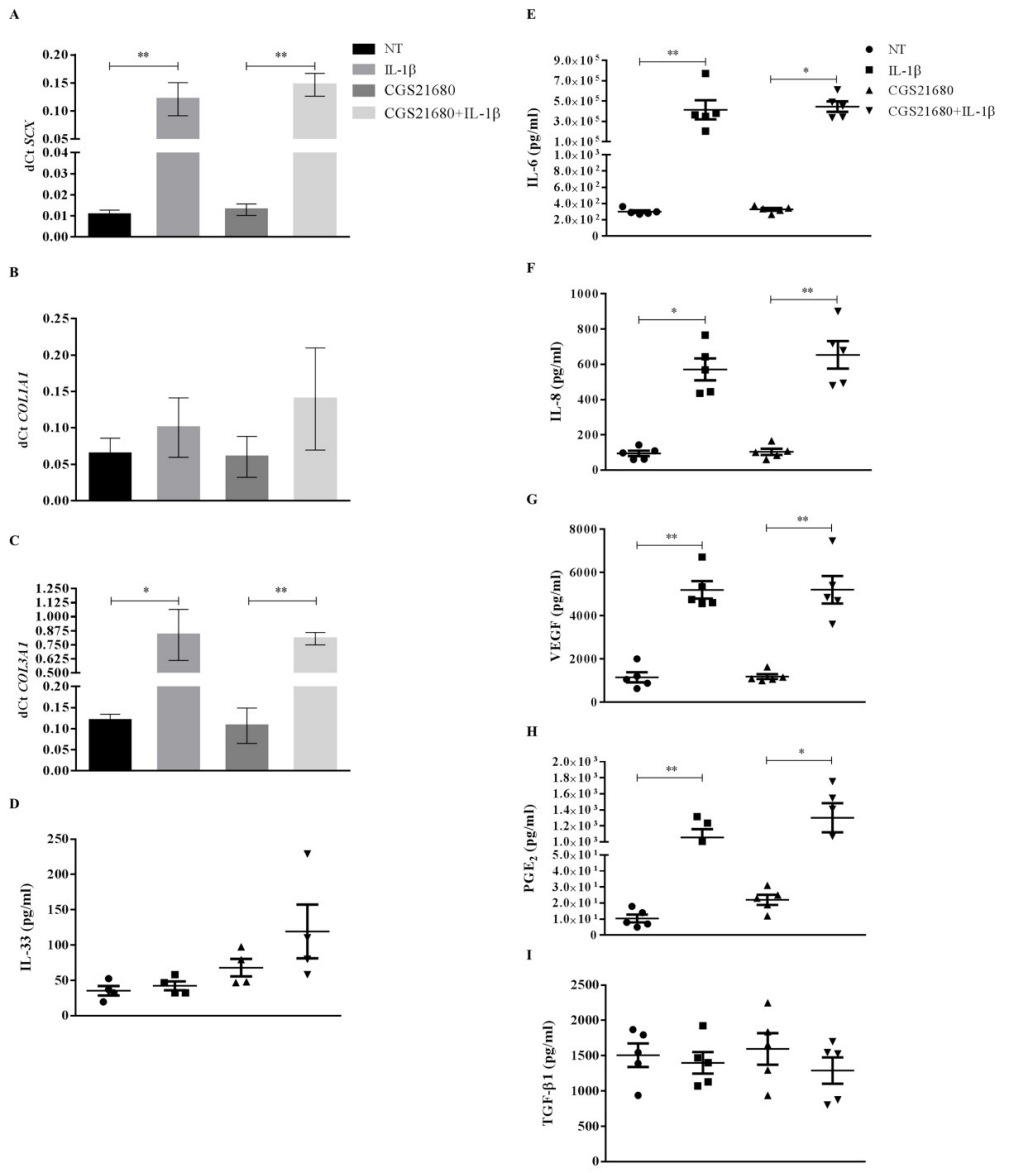
Gene expression and protein release of TCs after 48 hours exposure to IL-1β and/or CGS-21680. *SCX* (A), *COL1A1* (B) and *COL3A1* (C) expression, IL-33 (D), IL-6 (E), IL-8 (F), VEGF (G), PGE_2_ (H) and TGFβ (I) secretion. * p<0.05, ** p<0.01. n = 5. NT = not treated cells.

The inflammatory stimulus mediated by IL-1β induced an upregulation of *ADORA2A* expression in TCs (NT *vs* IL-1β p = 0.02, CGS-21680 *vs* CGS-21680*+*IL-1β p = 0.002) (Fig 3A), suggesting an involvement of this receptor in the anabolic response of these cells to the inflammation, with modulation of A_2A_ARs density, although not significant (Table 1, Fig 3B). In fact, an upregulation of the tenogenic markers *SCX* (NT *vs* IL-1β p = 0.006, CGS-21680 *vs* CGS-21680*+*IL-1β p = 0.009) and *COL3A1* (NT *vs* IL-1β p = 0.02, CGS-21680 *vs* CGS-21680*+*IL-1β p = 0.03) was observed (Fig 4A–4C). As expected, in this inflamed condition, TCs showed a concomitant secretion of the inflammatory IL-6 (NT *vs* IL-1β p = 0.005, CGS-21680 *vs* CGS-21680*+*IL-1β p = 0.01), PGE_2_ (NT *vs* IL-1β p = 0.004, CGS-21680 *vs* CGS-21680*+*IL-1β p = 0.01), inflammatory and pro-angiogenic IL-8 (NT *vs* IL-1β p = 0.01, CGS-21680 *vs* CGS-21680*+*IL-1β p = 0.004) and of the angiogenic VEGF (NT *vs* IL-1β p = 0.006, CGS-21680 *vs* CGS-21680*+*IL-1β p = 0.009), irrespective of the presence of CGS-21680 (Fig 4E–4H).

*COL1A1* expression and IL-33 and TGFβ secretion were not modulated by IL-1β treatment (Fig 4B, 4D and 4I).

### Slight anabolic and anti-inflammatory response of inflamed TCs to PEMF exposure

**Fig 5:**
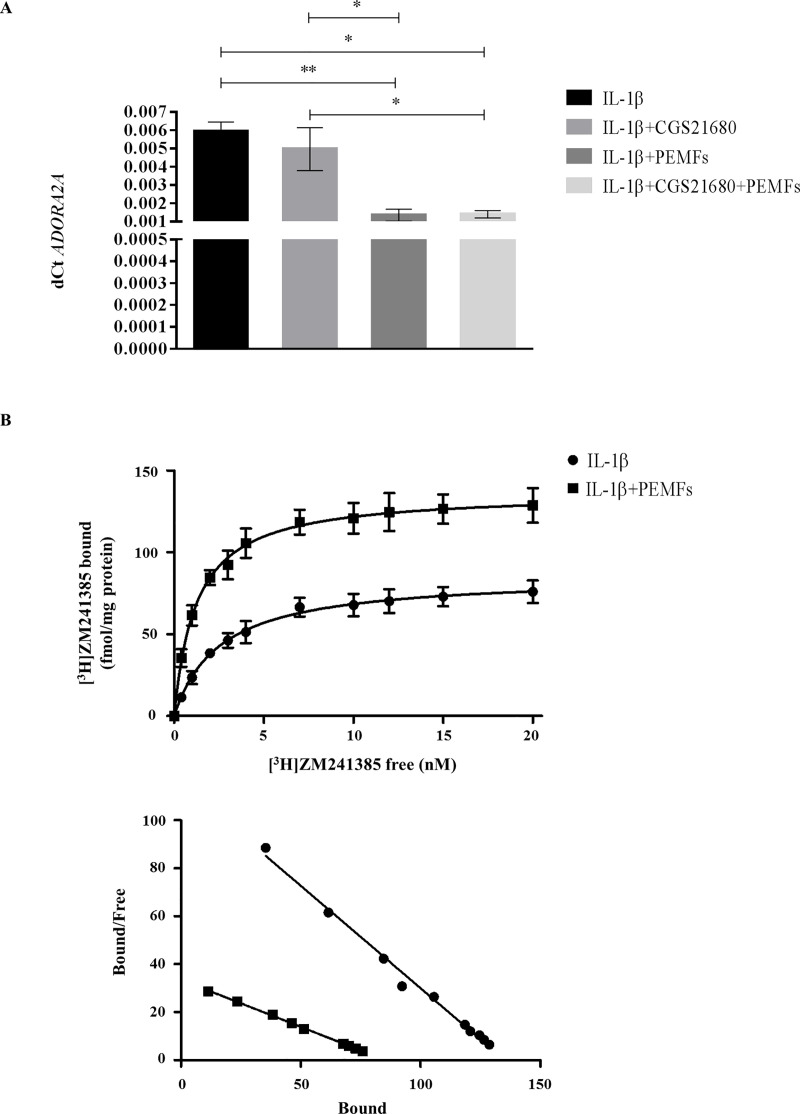
Exposure of TCs to IL-1β and PEMF for 48 hours. Expression of *ADORA2A* (A) and A_2A_ adenosine receptor saturation binding experiments (B). * p<0.05, ** p<0.01. n = 5.

**Fig 6:**
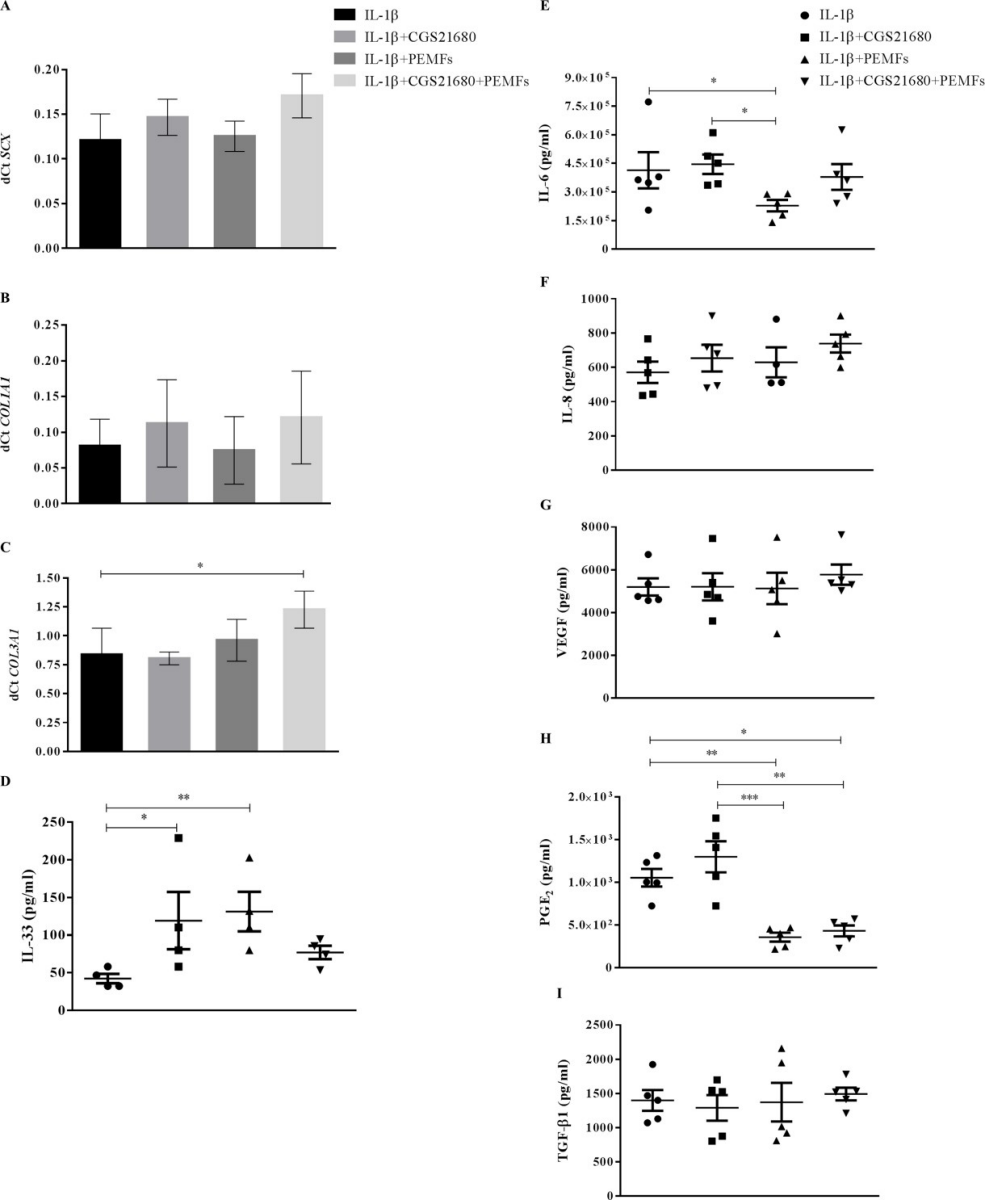
Gene expression and protein release of TCs after 48 hours exposure to PEMF and/or CGS-21680 in inflamed conditions. *SCX* (A), *COL1A1* (B) and *COL3A1* (C) expression, IL-33 (D), IL-6 (E), IL-8 (F), VEGF (G), PGE_2_ (H) and TGFβ (I) secretion. * p<0.05, ** p<0.01, *** p<0.001. n = 5.

When TCs cultured in presence of IL-1β were exposed to PEMFs, a downregulation of *ADORA2A* expression was observed (IL-1β and IL-1β+CGS-21680 *vs* IL-1β+PEMFs and *vs* IL-1β+CGS-21680+PEMFs p = 0.006, p = 0.01 and p = 0.02, p = 0.03, respectively) (Fig 5A), with modulation, although not significant, in A_2A_ARs density and ligand affinity (Table 1, Fig 5B). Only the combination of PEMF and CGS-21680 was able to significantly upregulate the expression of *COL3A1* (p = 0.04) (Fig 6C), whereas CGS-21680 (p = 0.02) and PEMF (p = 0.004) alone provoked an upregulation of the secretion of IL-33 with respect to unstimulated cells (Fig 6D). Oppositely, a slight downregulation of IL-6 was observed when IL-1β-treated cells were stimulated with PEMF (p = 0.04 and p = 0.01 *vs* IL-1β and IL-1β+CGS-21680, respectively) (Fig 6E); similarly and in a much more evident way the secretion of PGE_2_ was downregulated by PEMF, both in presence and absence of CGS-21680 (IL-1β and IL-1β+CGS-21680 *vs* IL-1β+PEMFs and *vs* IL-1β+CGS-21680+PEMFs p = 0.009, p = 0.04 and p<0.001, p = 0.006, respectively) (Fig 6H).

The anabolic markers *SCX*, *COL1A1*, TGFβ and the angiogenic markers IL-8, VEGF were not modulated by any treatment (Fig 6A, 6B, 6F, 6G and 6I).

Anti-inflammatory cytokines IL-10 and IL-1Ra resulted undetected in all the samples, independently from the presence of inflammatory (IL-1β), biophysical (PEMF) or biochemical (CGS-21680) stimulus, alone or combined.

Table 2 summarizes the modulation of the parameters evaluated after the 48 h exposure of TCs to PEMF in basal or inflamed conditions (+ IL-1β).

**Table 2:** Summary of parameters evaluated in TCs after PEMF treatment for 48 hours in basal or inflamed (+ IL1β) conditions.

	PEMF	
	*Basal*	*+ IL1β*
A_2A_AR density/affinity	+ (n.s.)	+ (n.s.)
*ADORA2A*	- (n.s.)	-
*SCX*	+	=
*COL1A1*	=	=
*COL3A1*	=	=
IL-33	+	+
IL-6	=	-
IL-8	=	=
VEGF	=	=
PGE_2_	=	-
TGF-β1	=	=
IL-1Ra	n.d.	n.d.
IL-10	n.d.	n.d.

+: significantly increase.

-: significantly decrease.

=: unchanged.

n.d.: not detected.

n.s.: non significant.

## Discussion

The main finding of this study is that A_2A_ARs have a role in the TC anabolic/reparative response to PEMFs. The response of TCs to PEMFs might depend on the higher A_2A_ARs surface exposition/ligand affinity, without a concomitant upregulation of the *ADORA2A* expression, which, on the contrary, was downregulated by the combined treatments of IL-1β and PEMFs, probably through a feedback mechanism.

This observation is in contrast with what observed in human osteoarthritic synovial fibroblasts, T/C-28a2 chondrocytes and hFOB 1.19 osteoblasts where PEMFs determined a significant upregulation of *ADORA2A* expression and protein production [25, 26].

TCs stimulated with PEMFs showed an early anabolic and reparative response that is typical of the physiological first inflammatory phase of tendon healing. Indeed, an increase of anabolic *SCX* and IL-33-mediated *COL3A1* expression, without *COL1A1* involvement was observed in presence of a higher secretion of the inflammatory cytokines IL-6 and PGE_2_. Differently from what already reported [19, 20], in the present work VEGF, TGFβ and *COL1A1* were not affected by any biophysical stimulation, either in presence or absence of receptor agonist, probably due to the different length of PEMF exposure. On the other hand, other authors showed no effect of PEMF on *COL1A1* and VEGF levels, although the experimental setting (waveform, frequency, length of exposure and magnetic field intensity) was very different from that used in this study [29]. Moreover, anti-inflammatory cytokines IL-10 and IL-1Ra were undetectable in all the samples. The release of IL-10 and TGFβ along with a reduced *COL3A1* expression was previously observed in TCs exposed to 1.5 mT-PEMFs for 8 and 12 hours [20]. This could be due to the fact that TCs are a heterogeneous population composed of both differentiated and undifferentiated cells [30, 31] and that their response to PEMFs likely depends on the length of PEMF exposure.

IL-1β is a well-known inflammatory stimulus, already used to test the PEMF anti-inflammatory potential in human synoviocytes [25] and in cell lines of chondrocytes and osteoblasts [26]. TCs showed a reparative profile when exposed to IL-1β. Also in this case, an increase in the expression of *SCX* and *COL3A1* along with a higher secretion of IL-6 and PGE_2_ was observed, confirming previously reported data [7, 9]. Moreover, angiogenesis was promoted as highlighted by the increase of IL-8 and VEGF secretion. Together with these observations, we also found a concomitant increase of *ADORA2A* expression following IL-1β treatment.

In human T/C-28a2 chondrocytes and hFOB 1.19 osteoblasts cultured in presence of IL-1β, the concomitant exposure to PEMFs and CGS-21680 determined a decrease of IL-6, IL-8, PGE_2_ and VEGF secretion [26]. In osteoarthritic synoviocytes in presence of IL-1β, PEMF exposure alone determined a decrease of IL-6 and PGE_2_ secretion and the inhibition of their release was equal to that induced in presence of CGS-21680 [25]. Similarly, in our study, treating TCs in presence of IL-1β with PEMFs, either alone or combined with CGS-21680, determined a significant reduction of PGE_2_. Moreover, we observed a slight anabolic response with an increase of IL-33 secretion and a mild downregulation of the inflammatory IL-6 secretion after PEMF treatment in comparison with cells treated with IL-1β alone or combined with CGS-21680, respectively. IL-8 was never modulated by PEMFs in TCs, synoviocytes and cell lines of chondrocytes and osteoblasts in presence of IL-1β. Likewise synoviocytes [25], also TCs showed no changes in the anti-inflammatory IL-10 secretion after PEMF treatment. The selective activation of A_2A_ARs through the use of CGS-21680 in presence of PEMFs determined a significant increase in the IL-10 release in synoviocytes [25]. This was not observed in TCs where IL-10 was always below the detectable level.

The main limitation of this study is that only one subgroup of ARs was included in the analysis. Nevertheless, since PEMFs have been described to have an effect on the increase of the A_2A_ and A_3_ AR expression, with the concomitant reduction of the release of inflammatory mediators [25, 26], the present investigation was performed on receptors of the subgroup more likely involved in inflammatory responses.

## Conclusion

In conclusion, our data suggest that the attempt of TCs to counteract the catabolic/inflammatory state induced by IL-1β is in part mediated by A_2A_ARs and reinforced by PEMF treatment. Although the clinical relevance is not direct, this investigation could be considered as the first attempt to clarify the effect of A_2A_ARs in the response of tendon cells to PEMF. Further experiments are required to tune the PEMF treatment protocols for tendon disorders, focusing particularly on the duration and intensity of the stimuli as well as on the best timing for application to avoid a feedback mechanism counteracting the potential therapeutic PEMF effects.
